# The long non-coding RNA PTTG3P promotes cell growth and metastasis via up-regulating PTTG1 and activating PI3K/AKT signaling in hepatocellular carcinoma

**DOI:** 10.1186/s12943-018-0841-x

**Published:** 2018-05-26

**Authors:** Jin-lan Huang, Shun-wang Cao, Qi-shui Ou, Bin Yang, Shi-hao Zheng, Jing Tang, Jing Chen, Yan-wei Hu, Lei Zheng, Qian Wang

**Affiliations:** 1 Laboratory Medicine Center, Nanfang Hospital, Southern Medical University, Guangzhou, 510515 Guangdong China; 2 Department of Clinical Laboratory, First Affiliated Hospital of Fujian Medical University, Fuzhou, Fujian China; 3 Department of Neurosurgery, Fujian Provincial Hospital, Fuzhou, Fujian China; 4 Department of Internal Medicine-Oncology, First Affiliated Hospital of Guangxi Medical University, Nanning, Guangxi China

**Keywords:** Hepatocellular Carcinoma, Long non-coding RNA, PTTG3P, PTTG1, PI3K/AKT signaling

## Abstract

**Background:**

Dysfunctions of long non-coding RNA (lncRNAs) have been associated with the initiation and progression of hepatocellular carcinoma (HCC), but the clinicopathologic significance and potential role of lncRNA PTTG3P (pituitary tumor-transforming 3, pseudogene) in HCC remains largely unknown.

**Methods:**

We compared the expression profiles of lncRNAs in 3 HCC tumor tissues and adjacent non-tumor tissues by microarrays. In situ hybridization (ISH) and quantitative real-time polymerase chain reaction (qRT-PCR) were applied to assess the level of PTTG3P and prognostic values of PTTG3P were assayed in two HCC cohorts (*n* = 46 and 90). Artificial modulation of PTTG3P (down- and over-expression) was performed to explore the role of PTTG3P in tumor growth and metastasis in vitro and in vivo. Involvement of PTTG1 (pituitary tumor-transforming 1), PI3K/AKT signaling and its downstream signals were validated by qRT-PCR and western blot.

**Results:**

We found that PTTG3P was frequently up-regulated in HCC and its level was positively correlated to tumor size, TNM stage and poor survival of patients with HCC. Enforced expression of PTTG3P significantly promoted cell proliferation, migration, and invasion in vitro, as well as tumorigenesis and metastasis in vivo. Conversely, PTTG3P knockdown had opposite effects. Mechanistically, over-expression of PTTG3P up-regulated PTTG1, activated PI3K/AKT signaling and its downstream signals including cell cycle progression, cell apoptosis and epithelial-mesenchymal transition (EMT)-associated genes.

**Conclusions:**

Our findings suggest that PTTG3P, a valuable marker of HCC prognosis, promotes tumor growth and metastasis via up-regulating PTTG1 and activating PI3K/AKT signaling in HCC and might represent a potential target for gene-based therapy.

**Electronic supplementary material:**

The online version of this article (10.1186/s12943-018-0841-x) contains supplementary material, which is available to authorized users.

## Background

Hepatocellular carcinoma (HCC) ranks as the fifth most common cancer in men and the seventh in women with annual incidence rates of ~ 750,000 worldwide [1, 2]. Despite curative improvements made in HCC therapy recent years, the 5-year survival rate of HCC subjects still remains poor due to the spread, metastases and high rate of recurrence [3, 4]. To date, HCC has become the second most frequent cause of cancer-related death [1]. Although many altered pathways and aberrantly expressed genes involved in hepatocarcinogenesis were identified, the precise molecular mechanisms for HCC are not entirely clear [2, 5]. Thus, in order to improve the prognosis of patients with HCC, an urgent need for novel molecular markers that can help in early diagnosis, risk assessment and therapy appears to be imperative.

Most of the eukaryotic genome is transcribed, yielding a complex network of transcripts. And those greater than 200 nt in length with limited or no protein-coding capacity are generally defined as long non-coding RNAs (lncRNAs [6]. LncRNAs have been shown to emerge as essential regulators in a diverse range of cellular functions, such as development, differentiation, and cell fate as well as tumorigenesis [7, 8]. Mounting evidence has linked mutation and dysregulation of lncRNAs to cancer initiation, growth and metastasis and they may act as oncogenic factors or tumor suppressors [8, 9]. Recently, several lncRNAs have been reported to participate in diverse biological processes involved in hepatocarcinogenesis, including cell proliferation, apoptosis, metastasis and angiogenesis [6, 10, 11]. Examples include DANCR [12], AFAP1-AS1 [13], UCA1 [14] and ZEB1-AS1 [15]. However, investigations on the function and clinical significance of the majority of HCC-related lncRNAs still remain limited.

In this study, we identified a novel lncRNA PTTG3P, termed pituitary tumor-transforming 3, pseudogene (NCBI Accession NO.NR_002734) via microarray analysis. The expression and localization of PTTG3P were analyzed by quantitative real time polymerase chain reaction (qRT-PCR) and in situ hybridization (ISH), respectively, using patient samples from 2 HCC cohorts. Our data showed that PTTG3P was frequently up-regulated in HCC and high levels of PTTG3P positively correlated with poor prognosis in patients with HCC. Further investigations on the role of PTTG3P and its molecular basis in HCC revealed that PTTG3P promoted cell growth, metastasis and tumorigenicity via targeting PTTG1 (pituitary tumor-transforming 1) and activating PI3K/AKT signaling pathway. These results indicated that PTTG3P harbors great potential significance as a prognostic biomarker and a therapy target for HCC.

## Methods

### Patient samples

Two independent cohorts involving 136 HCC patients were enrolled in this study. In cohort 1, fresh HCC samples and adjacent non-tumor tissues were obtained from 46 patients who had undergone routine surgery from 2012 to 2014 at Nanfang Hospital, Southern Medical University. Tissues were frozen and stored in liquid nitrogen until further use. In cohort 2, paraffin-embedded samples were obtained from 90 patients diagnosed as HCC between January 2007 and December 2009 at the same hospital. Medical records of all patients provided information of age, gender, and following parameters: liver cirrhosis, tumor size, tumor number, Edmonson grade and TNM stage. The patients in cohort 2 were followed up for 5 years. Written informed consent for the biological studies was obtained from each patient involved in the study, and the study was approved by the Ethics Committee of Nanfang Hospital.

### Microarray analysis

Total RNA from 3 HCC tumor tissues and paired non-tumor tissues were isolated using Trizol (Invitrogen, Carlsbad, CA). Total RNA was quantified by the NanoDrop ND-1000 and RNA integrity was assessed by standard denaturing agarose gel electrophoresis. For microarray analysis, Agilent Array platform was employed. Total RNA was amplified and transcribed into fluorescent cRNA using Agilent’s Quick Amp Labeling Kit. The labeled cRNAs were hybridized onto the Human LncRNA Array v2.0 (8 × 60 K, Arraystar). After having washed the slides, the arrays were scanned by the Agilent Scanner G2505C. Agilent Feature Extraction software (version 11.0.1.1) was used to analyze acquired array images. Quantile normalization and subsequent data processing were performed using the GeneSpring GX v12.0 software package (Agilent Technologies). After quantile normalization of the raw data, lncRNAs and mRNAs that at least 2 out of 6 samples have flags in Present or Marginal (“All Targets Value”) were chosen for further data analysis. Differentially expressed lncRNAs and mRNAs with statistical significance between the two groups were identified through Volcano Plot filtering. Pathway analysis and GO analysis were applied to determine the roles of these differentially expressed mRNAs played in these biological pathways or GO terms. Finally, hierarchical clustering was performed to show the distinguishable lncRNAs and mRNAs expression pattern among samples.

### Cell culture

The HepG2 and Hep3Bcell lines were obtained from the Cell Bank of Type Culture Collection (Chinese Academy of Sciences, Shanghai, China). Cells were maintained in Dulbecco’s modified Eagle’s medium (DMEM, Gibco, Gaithersburg, MD,USA) supplemented with 10%fetal bovine serum (FBS, Gibco)and incubated at 37°Cin an atmosphere of 5% CO2.

### RNA extraction and quantitative real-time PCR analysis (qRT-PCR).

RNA extraction and qRT-PCR were performed as described previously [16, 17]. The primers used are presented in Additional file 1: Table S1.

### Western blot analysis

Western blotting was performed using a SDS-PAGE Electrophoresis System according to the previous description [16, 17] with antibodies specific for C-myc (Cell Signaling Technology, Beverly, MA, USA), CDK4 (Cell Signaling Technology), CDK6(Cell Signaling Technology), CyclinD1(Cell Signaling Technology), Rb (Cell Signaling Technology), p-Rb (Cell Signaling Technology), Caspase3 (Immunoway, USA), Cleaved Caspase3 (Cell Signaling Technology), Snail (Proteintech, USA), Slug (Proteintech), E-cadherin (Cell Signaling Technology), N-cadherin (Cell Signaling Technology), PI3K (Abclonal Technology), p-PI3K(Cell Signaling Technology), AKT(Cell Signaling Technology), p-AKT(Cell Signaling Technology), PTTG1(Cell Signaling Technology), β-Tublin (Cell Signaling Technology) and β-actin (Proteintech).Signals were detected using enhanced chemiluminescence reagents (Millipore, Schwalbach/Ts., Germany).

### Construction of stable cell lines

To obtain cell lines stably over-expressing PTTG3P, HepG2 and Hep3B cells were infected with the Lv-PTTG3P and Lv-con viruses (LAND, Guangzhou, China). To observe the knockdown effects of PTTG3P, HepG2 and Hep3B cells were transfected with the shRNA-PTTG3P (sh-PTTG3P)or control (sh-con) viruses purchased from GeneChem (Shanghai, China). The infection efficiency was confirmed by qRT–PCR.

### Cell counting kit-8 (CCK-8) assay, 5-ethynyl-2′-deoxyuridine (EdU) incorporation assay, Colony formation assay and Cell cycle analysis.

CCK-8 assay, EdU incorporation assay, Colony formation assay and Cell cycle analysis were performed as described in previous study [16].

### Cell apoptosis analysis

The apoptosis assay was done with the AnnexinV-7AAD apoptosis detection kit (KeyGEN BioTECH, Nanjing, Jiangsu Province, China) according to the manufacturer’s instructions. To detect the effect of PTTG3P over-expression on 5-FU-induced cell apoptosis, Lv-PTTG3P and Lv-con cells were seeded in 6-well plates and treated on the following day with 5-FU. After incubation for 48 h, cells were harvested, stained using AnnexinV-7AAD apoptosis detection kit and then analyzed by FACS cytometry (BD Biosciences, San Jose, CA, USA).All experiments were performed in duplicate and reproducibility was checked in three independent experiments.

### Cell migration and invasion assays

Cell migration and invasion assays were performed with Boyden chambers (pore size: 8 μm, 24-well; BD Biosciences) with Matrigel (for the invasion assay)or without Matrigel (for the migration assay) following the manufacturer’s protocol. For cell migration assays, cells were detached and washed with PBS, resuspended in serum-free medium, and 200 μl of cell suspensions (a total of 5 × 10^4^ cells) was added to the upper chamber. Medium with 20% FBS was added to the bottom wells of the chambers. The cells that had not migrated were removed from the upper face of the filters using cotton swabs, and the cells that had migrated to the lower face of the filters were fixed with fixed with methanol, stained with crystal violet solution, photographed under microscope and quantified. The mean of triplicate assays for each experimental condition was used. Similar inserts coated with Matrigel were used to determine the invasive potential in the invasion assay.

### Animal studies

All animal studies were approved by the Animal Experimental Committee of Nanfang Hospital. The male BALB/C nude mice (4–5 weeks old) were purchased from the Experimental Animal Center of Southern Medical University, bred and maintained in a specific pathogen-free facility. For in vivo tumor growth assays, a total of 1 × 10^7^ HepG2 cells stably transfected with Lv-PTTG3P or Lv-con, sh-PTTG3P or sh-con in 100 μl DMEM medium was independently injected subcutaneously into the left back and right back of 10 nude mice. Tumor volumes were monitored and calculated bylength×width^2^ × 0.5 weekly after implantation. All mice were sacrificed four weeks later. Mean percent of body weight (±SEM) and tumor size for each group was measured. For in vivo tumor metastasis assays, 1 × 10^7^HepG2/sh-PTTG3P and HepG2/sh-con cells were injected into nude mice through the spleen, respectively. Two months later, all mice were sacrificed to observe the tumor metastasis in liver and abdominal cavity. The metastatic tissues were photographed and analyzed by H&E staining.

### In situ hybridization

The ISH probe used for detecting PTTG3P-labeled digoxin was designed and synthesized by Exiqon (Shanghai, China). ISH was performed using the ISH Kit (Boster Bio-Engineering Company, Wuhan, China) and the stained tissue sections were reviewed and scored separately by two pathologists blinded to the clinical parameters. The score standard for the staining intensity was as follows: 0 (negative), 1 (weak), 2(medium), and 3 (strong); and 0 (0%), 1 (1–25%), 2(26–50%), 3 (51–75%), and 4 (76–100%) for the staining extent. For statistical analysis, a final staining score of 3 or higher was considered to be high expression, respectively.

### Statistical analysis

SPSS 13.0 software (SPSS Inc., Chicago, IL, USA) and GraphPad prism 5 software (GraphPad Software, Inc., La Jolla, CA, USA) were used to analyze all data for statistical significance. The Chi-Square test was applied to the examination of relationship between lncRNAPTTG3P levels and clinicopathological characteristics. Survival curves were plotted by Kaplan–Meier method and log rank test. The significance of various survival-related variables was assessed by Cox regression model in the multivariate analysis. Two-tailed Student’s t-test was used for comparisons of two independent groups. Repeated measurement data analysis of variance was performed for results from CCK-8 assays and tumor growth curve determinations. Statistical significance was set at *P < 0.05.

## Results

### LncRNA expression profile in HCC

**Fig. 1:**
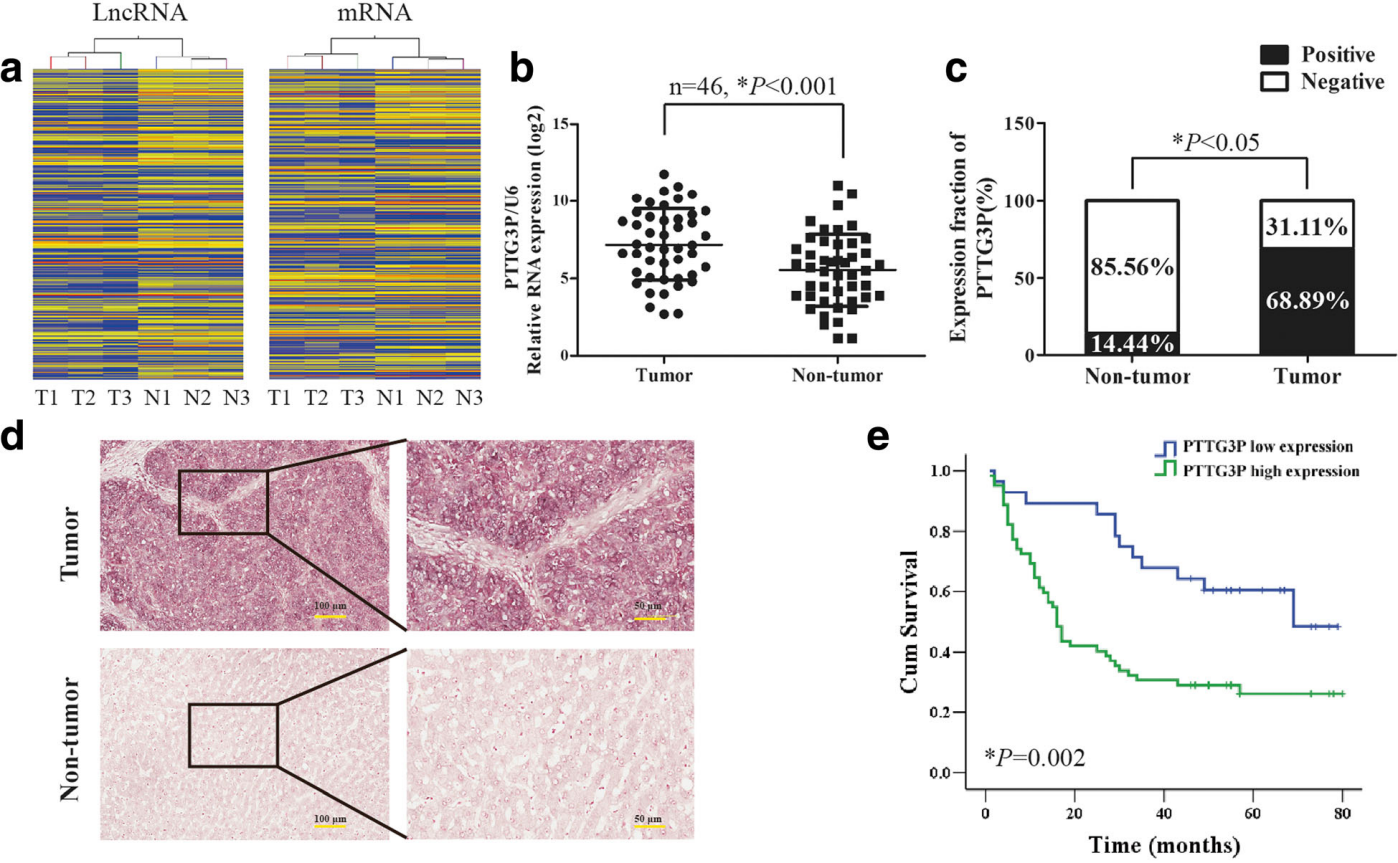
LncRNA PTTG3P is frequently up-regulated in HCC. (**a**) Hierarchical clustering analysis of 884 differentially expressed lncRNAs and 979 differentially expressed mRNAs between 3 HCC tumor tissues and paired non-tumor tissues. Up-regulation is shown in yellow and down-regulation is in blue. T, tumor tissues; N, non-tumor tissues. (**b**) The level of lncRNA PTTG3P was detected in 46 HCC tumor tissues and paired non-tumor tissues by qRT-PCR.U6 was used as a housekeeping gene. (**c**) The levels of PTTG3P in HCC tissues and adjacent non-tumor tissues was evaluated by ISH assays (cohort 2, *n* = 90). (**d**) Representative images of lncRNA PTTG3P expression from HCC tumor tissues and non-tumor tissues by ISH assays. (**e**) Kaplan-Meier survival analysis of overall survival in 90 patients with HCC (cohort 2) according to PTTG3P expression. Accumulation expression of PTTG3P was unfavorable for HCC prognosis. The log-rank test was used to calculate *P* values.*P < 0.05

To identify transcripts involved in hepatocarcinogenesis, lncRNAs and mRNAs expression profiles were determined by microarray analysis. The data are accessible through GeneExpression Omnibus Series accession number GSE89186 (Additional file 2: Table S2). Comparison between HCC tumor tissues and adjacent normal tissues revealed that approximately 884 lncRNAs (335 up-regulated and 549 down-regulated) and 979 mRNAs (449 up-regulated and 530 down-regulated) were differentially expressed (fold change> 1.5, *P* < 0.05). Hierarchical clustering of these lncRNAs and mRNAs based on centred Pearson correlation clearly separated HCC tissues from adjacent normal tissues (Fig. 1a).Taking the mRNAs of this profile as input, the pathway analysis revealed that cell cycle was the most affected biological process (Additional file 3: Figure S1a). The microarray analysis revealed a set of lncRNAs which were dysregulated in HCC tissues (Additional file 4: Table S3), of which lncRNAPTTG3P was one of the most up-regulated.

### LncRNA PTTG3P was frequently up-regulated in HCC

To confirm the frequent up-regulation of lncRNA PTTG3P in HCCs, we further examined the expression of PTTG3P by qRT-PCR in an additional 46 pairs of HCC/non-tumor tissues (cohort 1). Consistent with the observation in the lncRNA profiling study, the level of PTTG3P was significantly higher in tumor tissues compared with that in adjacent non-tumor tissues (*P* < 0.05, paired Student t test, Fig. 1b). Further ISH studies of PTTG3P were performed in 90 paraffin-embedded HCC tumor tissues and adjacent non-tumor tissues (cohort 2). High level of PTTG3P was found in 68.89% (62 of 90) of HCC tissues, compared with only 14.44% (13 of 90) of adjacent non-tumor tissues(*P* < 0.001)(Fig. 1c and d). The ISH studies also suggested PTTG3P to be mainly localized to the cytoplasm of HCC tissues (Fig. 1d).The level of PTTG3P in seven HCC cell lines (HepG2, Huh7, MHCC-97H, SMMC-7721, QGY7701, SK-Hep1, Hep3B) and hepatic immortal cell line LO2 was also detected by qRT-PCR (Additional file 3: Figure S1b).

### Over-expression of lncRNA PTTG3P is correlated with the progression and poor prognosis of HCC

We further investigated the associations between PTTG3P and clinicopathological characteristics in patients with HCC in cohort 1 and cohort 2. Chi-square test showed that the levels of PTTG3P significantly correlated with tumor size (*P* = 0.011) and TNM stage (*P* = 0.003) in cohort 2 (Table 1). The association between tumor size and PTTG3P expression was also confirmed by analysis of samples in cohort 1(*P* = 0.041, Table 2). Furthermore, Kaplan–Meier survival analysis indicated that HCC patients with higher levels of PTTG3P had a worse outcome (*P* = 0.002, Fig. 1e). Multivariate survival analysis indicated that the PTTG3P expression, TNM stage were 2 independent prognostic factors for outcomes in patients with HCC (Table 3).

**Table 1:** Correlation between lncRNA PTTG3P expression and clinicopathological characteristics in 90 HCC patients (cohort 2)

Features	All cases	lncRNA PTTG3P expression		^a^*P* value
		Low	High	
Total number	90	28	62	
Age				0.327
> 55	39	10	29	
≤55	51	18	33	
Gender				1.000
Male	75	23	52	
Female	15	5	10	
Liver cirrhosis				0.123
with	33	7	26	
without	57	21	36	
Tumor size(cm)				0.011*
> 5	53	11	42	
≤5	37	17	20	
Tumor number				1.000
solitary	84	26	58	
multiple	6	2	4	
Edmondson grade				0.550
I + II	57	19	38	
III	33	9	24	
TNM stage				0.003*
I + II	43	20	23	
III + IV	47	8	39	

^a^For analysis of correlation between lncRNA PTTG3P levels and clinical features, Pearson’s chi-square tests were used. When the expected count of variable was less than 5, Fisher’s exact tests was used. *, *P* < 0 .05

**Table 2:** Correlation between lncRNA PTTG3P expression and clinicopathological characteristics in 46 HCC patients (cohort 1)

Features	All cases	lncRNA PTTG3P expression		^a^*P* value
		Low	High	
Total number	46	16	30	
Age				0.057
> 55	20	10	10	
≤55	26	6	20	
Gender				0.698
Male	37	12	25	
Female	9	4	5	
HBsAg				0.605
positive	31	10	21	
negative	15	6	9	
Liver cirrhosis				0.382
with	27	8	19	
without	19	8	11	
Tumor size(cm)				0.041*
> 5	25	5	20	
≤5	23	11	12	
Tumor number				0.694
solitary	38	14	24	
multiple	8	2	6	
Edmondson grade				0.681
I + II	7	3	4	
III	39	13	26	

^a^For analysis of correlation between lncRNA PTTG3P levels and clinical features, Pearson’s chi-square tests were used. When the expected count of variable was less than 5, Fisher’s exact tests was used. *, *P* < 0 .05

**Table 3:** Univariate and multivariate analysis of overall survival in 90 patients with HCC by cox regression analysis

Variables	Univariate analysis			Multivariate analysis		
	HR	95% CI	*P* value	HR	95% CI	*P* value
Age	0.988	0.963–1.014	0.365	–	–	–
Gender	0.556	0.252–1.230	0.148	**–**	**–**	–
Liver cirrhosis	0.905	0.524–1.562	0.720	–	–	–
Tumor number	1.180	0.425–3.275	0.750	–	–	–
Edmonson grade	1.320	0.777–2.242	0.305	–	–	–
Tumor size	2.395	1.346–4.265	0.003*	1.383	0.692–2.763	0.359
TNM stage	3.042	1.729–5.351	< 0.001*	2.212	1.124–4.354	0.022*
PTTG3P expression	2.652	1.395–5.039	0.003*	2.131	1.107–4.103	0.024*

Abbreviations: *HR* hazard ratio, *CI* confidence interval, *, *P* < 0 .05

### LncRNA PTTG3P promotes cell proliferation in vitro and tumor growth in vivo

**Fig. 2:**
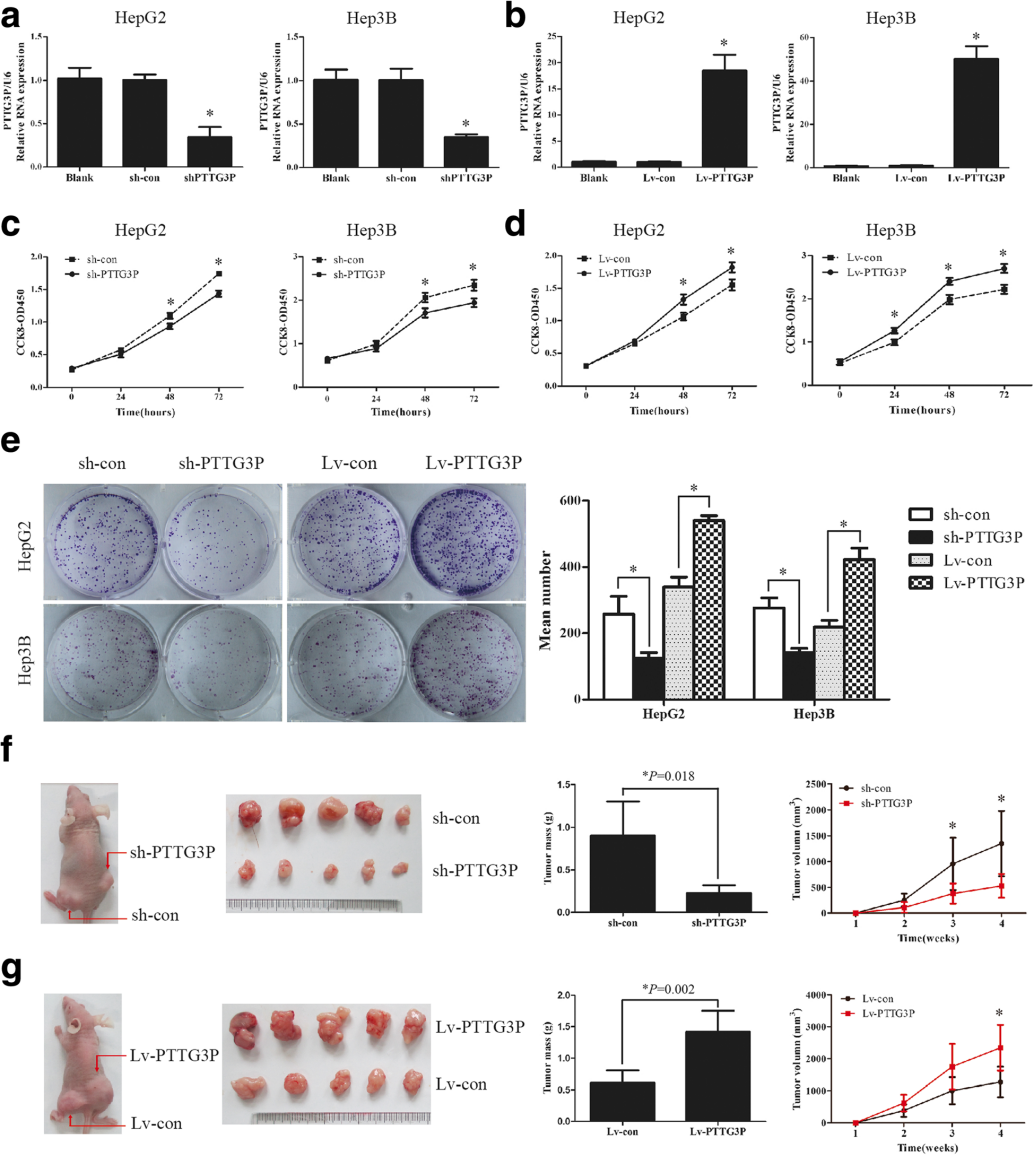
Over-expression of PTTG3P accelerates HCC cell growth in vitro and in vivo. (**a**) Knockdown of endogenous PTTG3P in specific shRNA transduced HepG2 and Hep3B cells. U6 was used as a housekeeping gene for qRT-PCR. (**b**) HepG2 and Hep3B cells were infected with lentivirus carrying the PTTG3P gene. The level of PTTG3P was significantly increased in HepG2 and Hep3B cells over-expressing PTTG3P when compared with control cells. U6 was used as a housekeeping gene for qRT-PCR. (**c**) After knockdown of PTTG3P in HepG2 and Hep3B cells, the cell viability was assessed by CCK-8 assays daily for 3 days. (**d**) Ectopic expression of PTTG3P promotes cell growth as determined by CCK-8 assays. (**e**) The effects of PTTG3P on cellular survival were assessed by colony formation assays. Colonies are shown in purple post staining with crystal violet (left). (**f**) Effects of PTTG3P over-expression on tumorigenesis in vivo. Representative images of tumors formed in nude mice injected subcutaneously with PTTG3P–silencing HepG2 cells were shown. The tumor mass were measured. The tumor volume was periodically tested for each mouse and tumor growth curve was plotted. (**g**) Effects of blocked PTTG3P expression on tumorigenesis in vivo. Red arrows indicate HCC tumors. Error bars represent mean ± SD from 3 independent experiments. *, *P* < 0.05

To gain insight into the biological role of PTTG3P in HCC, lentiviral shRNA vectors were used to specifically and stably knock down the endogenous expression of PTTG3P in HepG2 and Hep3B cells. Transfection with sh-PTTG3P constructs reduced PTTG3P expression by~ 65% compared with controls (Fig. 2a). CCK-8 assays revealed that depletion of PTTG3P expression caused evident compromised viability in both HepG2 and Hep3B cells (Fig. 2c).These results were validated in colony formation assays, which showed that sh-PTTG3P cells formed much less colonies than that of sh-con cells (Fig. 2e). To further confirm the effect of PTTG3P on cell proliferation and viability in HCC, we constructed HepG2 and Hep3B cells stably over-expressing PTTG3P by lentivirus infection (Fig. 2b). CCK-8 and colony formation assays indicated that over-expression of PTTG3P resulted in enhanced cell proliferation in both HepG2 and Hep3B cells (Fig. 2d and e). To further confirm the growth-enhancing effect of PTTG3P in vivo, HepG2 cells stably expressing sh-PTTG3P or sh-con, Lv-PTTG3P or Lv-con were subcutaneously injected into nude mice for xenoplantation. Xenograft tumors grown from cells with silenced PTTG3P expression had smaller mean volumes and weights than those grown from control cells (Fig. 2f and Additional file 5: Figure S2). Oppositely, PTTG3P over-expression induced tumor growth (Fig. 2g and Additional file 5: Figure S2). Thus, our results indicate that PTTG3Ppromotes cell proliferation in vitro and tumor growth in vivo.

### LncRNA PTTG3P induces cell-cycle progression and inhibits cell apoptosis in vitro

**Fig. 3:**
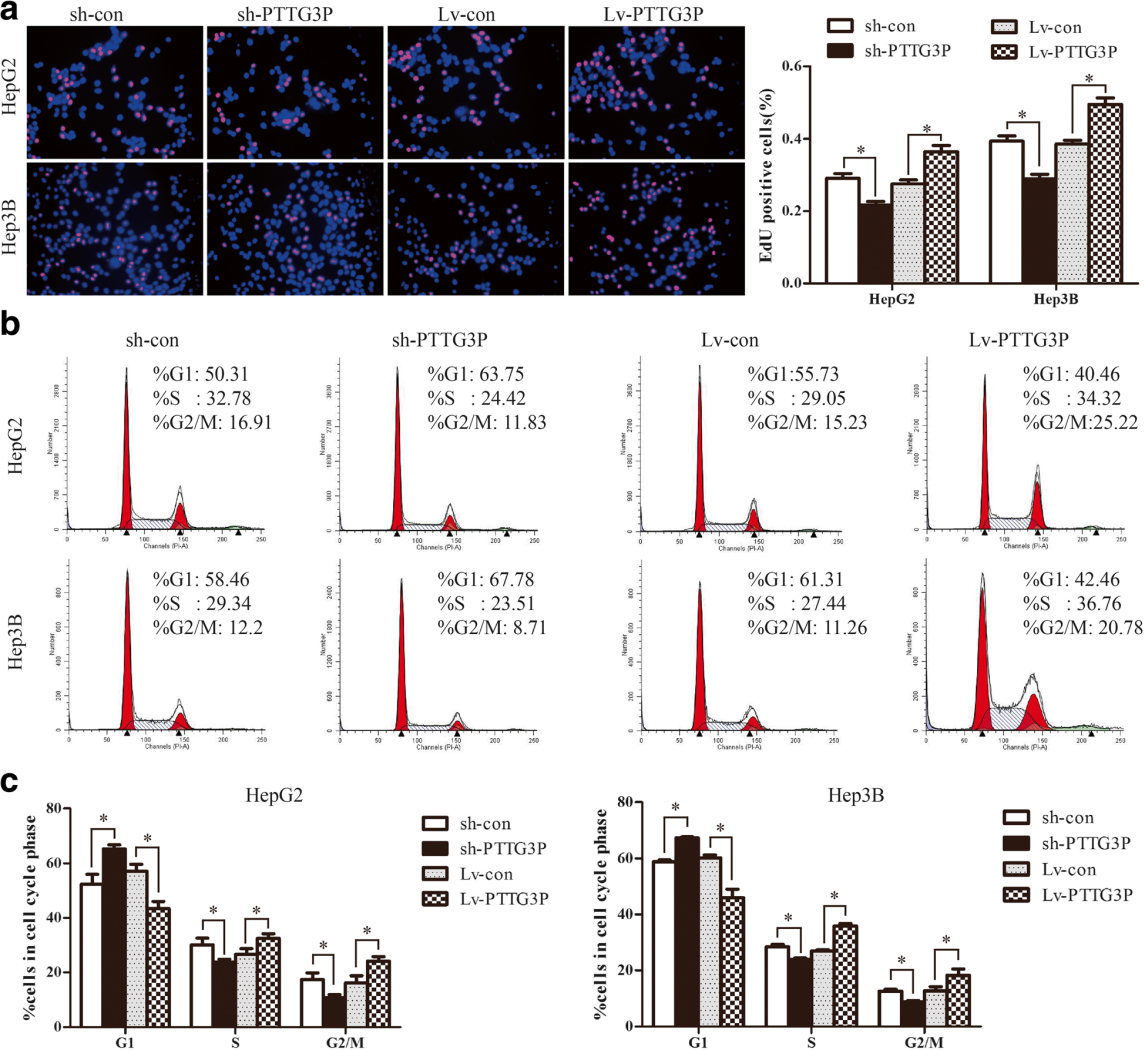
LncRNAPTTG3P induces cell-cycle progression in HCC cells. (**a**) HepG2 and Hep3B cells with enhanced or silenced PTTG3P expression were seeded on 96-well plates, and cell proliferation was examined by EdU immunofluorescence staining. The bar graph on the right shows the percentage of EdU-positive nuclei. (**b**) FACS analysis of HepG2 and He3B cells with elevated or blocked PTTG3P expression. (**c**) Proportion of cells in various phases of the cell cycle in HepG2 and Hep3B cells. Data are presented as mean ± SD for at least three independent experiments.*, *P* < 0.05

To uncover the possible mechanism of PTTG3P in controlling HCC cell proliferation, we further measured the cell cycle distribution by EdU incorporation assays and fluorescence-activated cell sorting (FACS). As shown in Fig. 3a, EdU-labelled cells was significantly decreased in HepG2 and Hep3B cells following PTTG3P knockdown whereas over-expression of PTTG3P resulted in a marked increase in the percentage of EdU positive cells in comparison with control cells, indicating that S-phase entry is facilitated by PTTG3P. Consistent with EdU results, knockdown of PTTG3P significantly increased the percentage of HepG2 cells inG1 phase from 52.38 to 65.37% compared with control, and decreased the percentage of HepG2 cells in S and G2/M phases (Fig. 3b and c).Similarly, stably blocked PTTG3P expression in Hep3B cells mainly led to a G1 accumulation and a decrease of S and G2/M phase(Fig. 3b and c).On the contrary, a reduction in the G1 population and an increase in the S and G2/M population were observed in HepG2 and Hep3B cells over-expressing PTTG3P(Fig. 3b and c). These results support a role of PTTG3P in inducing G1/S transition.

**Fig. 4:**
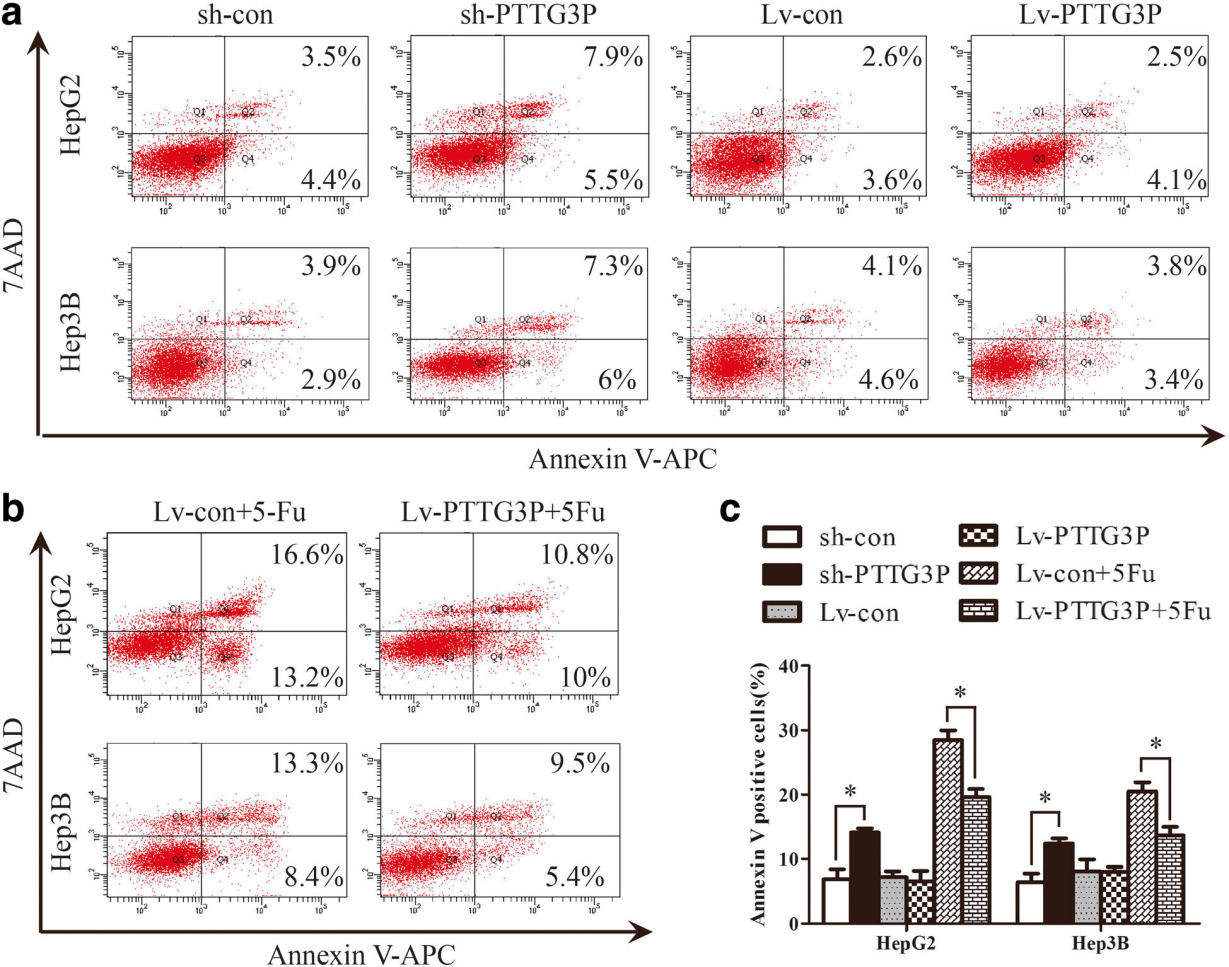
LncRNAPTTG3P inhibits cellular apoptosis of HCC cells. (**a**) HepG2 and Hep3B cells with enhanced or silenced PTTG3P expression were stained with a combination of annexin V and 7-AAD and analyzed by FACS. Cells positive for annexin V staining were counted as apoptotic cells. (**b**) The HepG2 and Hep3B cells over-expressing PTTG3P were treated with 5-fluorouracil for 48 h, stained with a combination of annexin-V and 7-AAD, and analyzed by flow cytometry. (**c**) The bar graph showed the percentage of apoptotic cells. The results are means of 3 independent experiments ± SD from 3 independent experiments. *, *P* < 0.05

Since the obligate compensatory suppression of apoptosis together with deregulated cell proliferation propelled the tumor cell and its progeny into uncontrolled expansion [18], we also detected the effect of PTTG3P on HCC cell apoptosis by FACS-based Annexin-V/7-AAD double staining. The results revealed that depletion of PTTG3P elevated the percentage of Annexin V–positive cells from 6.83 to 14.13% inHepG2 cells and from 6.4 to 12.4% in Hep3B cells relative to control cells (Fig. 4a). Intriguingly, over-expression of PTTG3P did not result in measurable changes in the apoptotic states of HepG2 and Hep3B cells (Fig. 4a, data not shown). However, it do have a protective effect on HepG2 and Hep3B cells treated with 5-fluorouracil (Fig. 4b and c).

### LncRNA PTTG3P promotes cell migration and invasion in vitro and tumor metastasis in vivo

**Fig. 5:**
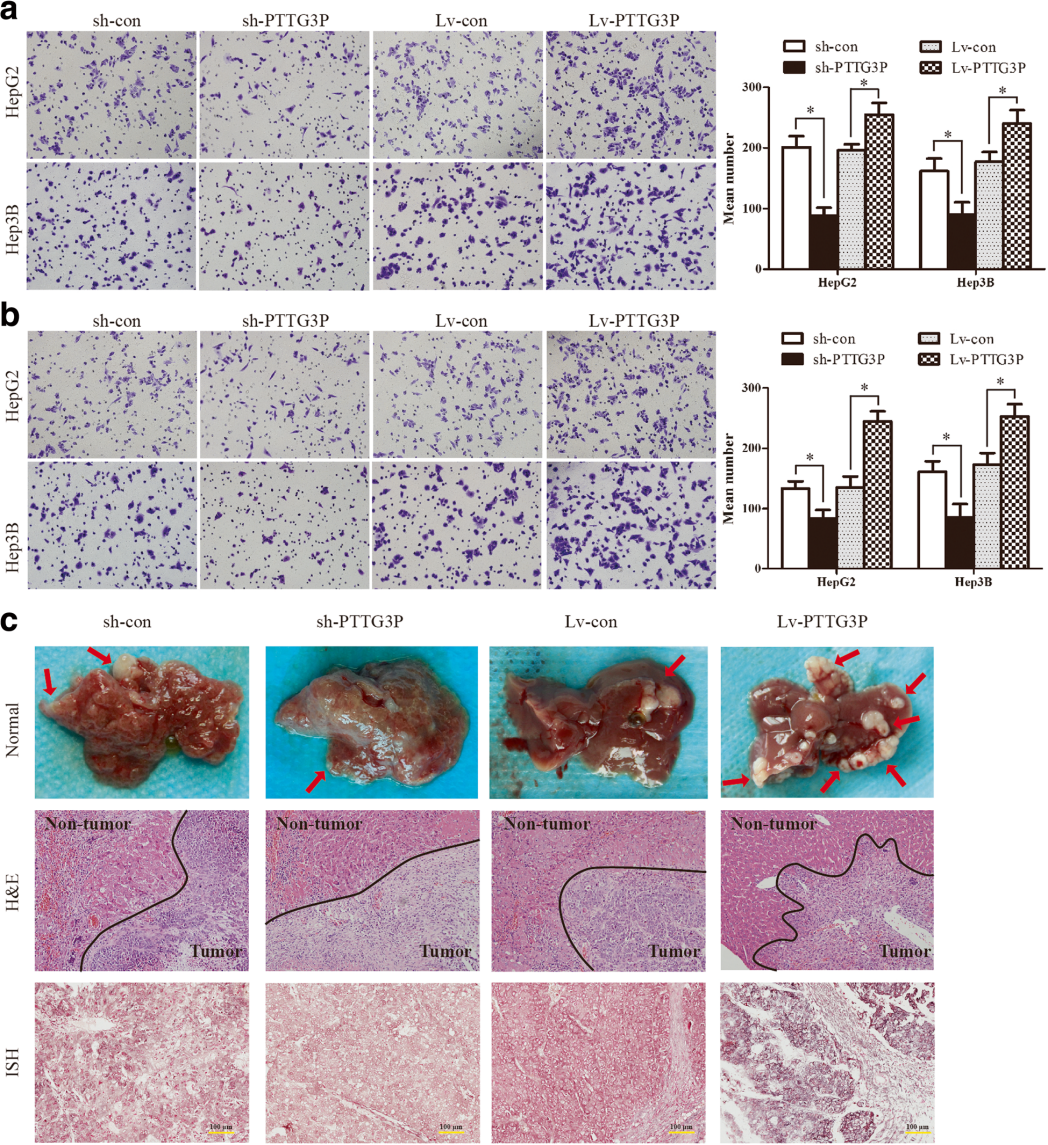
LncRNA PTTG3P promotes HCC cell metastasis in vitro and in vivo. (**a**) Transwell chamber assays showed that knockdown of PTTG3Preduced the migration ability while over-expression of PTTG3P enhanced the migration ability of HepG2 and Hep3B cells. (**b**) Boyden chamber assays revealed that stably suppressed PTTG3P expression inhibited invasiveness of HepG2 and Hep3B cells in vitro, whereas elevated PTTG3P expression had the opposite effects. (**c**) Representative images of hepatic metastasis tumors were obtained 40 days after spleen injection with sh-con, sh-PTTG3P, Lv-con or Lv-PTTG3P HepG2 cells, respectively(upper panel). H&E staining of metastastic tumor tissues were shown (middle panel). The boundary between tumor and normal tissue was indicated by black line. The level of PTTG3P in metastastic tumor tissues was assessed by ISH assays (lower panel). All values shown are mean ± SD of triplicate measurements and have been repeated 3 times with similar results. Independent t test was used to determine the differences between two groups.*, *P* < 0.05

To determine the effect of PTTG3P on cell migration and invasion, trans-well chamber and Boyden chamber assays were performed in HepG2 and Hep3B cells. Trans-well chamber assays showed that suppression of PTTG3P resulted in significantly diminished migratory potential in both HepG2 and Hep3B cells compared with control cells (Fig. 5a). Conversely, a marked increase of cell migration ability was observed in cells with elevated PTTG3P expression (Fig. 5a). For Boyden chamber assays, the cells were detached and subsequently added onto Matrigel-coated BD Falcon Cell Culture Inserts inside BD BioCoat Matrigel Invasion Chambers. Quantification of cells which had invaded through Matrigel, showed that knockdown of PTTG3P decreased the number of invaded cells in both HepG2 and Hep3B cells whereas over-expression of PTTG3P had opposite effects(Fig. 5b).Thus, these data suggest that lncRNA PTTG3P induces HCC cell migration and invasion in vitro. To determine its role on tumor metastasis in vivo, we further inoculated HepG2 cells intrasplenically into nude mice. As illustrated in Fig. 5c, depletion of PTTG3P reduced the liver metastases burden whereas over-expression of PTTG3P resulted in greater liver metastases burden. Moreover, H&E-stained liver sections revealed that control tumors showed extensive evidence of invasion into adjacent tissue, whereas PTTG3P-knockdown tumors maintained a distinct tumor–stroma boundary (Fig. 5c). Collectively, these data suggest PTTG3P promotes cell migration and invasion in vitro and tumor metastasis in vivo.

### LncRNA PTTG3P modulates the expression of multiple genes involved in cell cycle, cell apoptosis and EMT: PI3K/AKT activation in HCC cells was involved

**Fig. 6:**
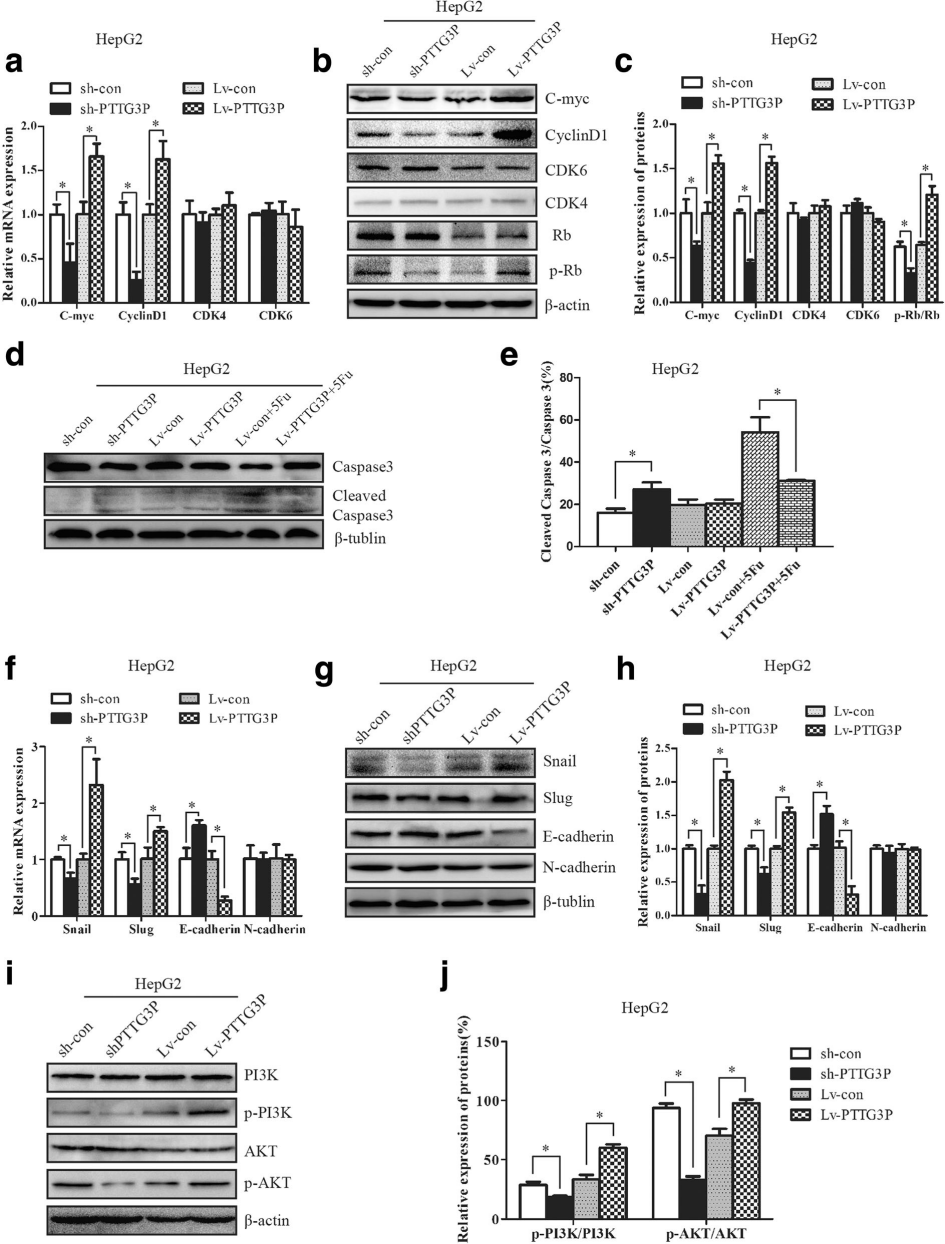
LncRNA PTTG3P regulates the level of multiple genes involved in cell cycle, cell apoptosis and EMT through PI3K/AKT pathway in HCC cells. (**a**-**c**) The relative levels of cell cycle associated genes, including C-myc, CyclinD1, CDK6, CDK4, Rb and phosphorylated Rb (p-Rb), were detected in HepG2 cells overexpressing PTTG3P and HepG2 cells with stably suppressed PTTG3P expression by qRT-PCR (**a**) and western blot with quantitative analysis (**b** and **c**). β-actin was used as a housekeeping gene for qRT-PCR and an internal control for western blotting analysis. (**d**-**e**)Western blot analysis of the levels of Caspase3 and cleaved Caspase3 in HepG2 cells with silenced or enhanced PTTG3P. β-tublin was used as an internal control. (**f**-**h**) Knockdown of endogenous PTTG3P in HepG2 cells reduced the mRNA (**f**) and protein levels (**g** and **h**) of several EMT-marker genes including Snail and Slug but enhanced E-cadherin expression. In contrast, the level of Snail and Slug increased and the mRNA (**f**) and protein (**g** and **h**) level of E-cadherin decreased in HepG2 cells with elevated PTTG3P expression. β-actin was used as a housekeeping gene for qRT-PCR. β-tublin was used as an internal control for western blot analysis. (**i**-**j**) The levels of PI3K, phosphorylated PI3K (p-PI3K), AKT, phosphorylated AKT (p-AKT) were examined by western blot analysis in HepG2 cells with silenced or enhanced PTTG3P. β-actin was used as an internal control. The experiments were performed in triplicate; the data are expressed as the mean ± SD. *, *P* < 0.05

To better understand the mechanism by which PTTG3P regulates cell cycle progression, cell apoptosis and metastasis, we examined the expression of several key regulators involved in these biological processes. Higher levels of oncogenic cell-cycle regulators including C-myc, CyclinD1 and p-Rb were observed in HepG2 cells with enhanced PTTG3P expression while knockdown of PTTG3P resulted in decreased levels of C-myc, CyclinD1 and p-Rb (Fig. 6a, b and c). Interestingly, neither PTTG3P over-expression nor suppression did affect the levels of CDK4, CDK6 and total Rb in HepG2 cells (Fig. 6a, b and c).Moreover, the expression of 2 well-defined apoptosis protein markers, caspase 3 and its active form of cleaved caspase 3, was detected by western blot analysis. The ratio of cleaved caspase 3/caspase 3 was remarkably elevated in HepG2 cells with blocked PTTG3P expression (Fig. 6d and e). Interestingly, no measurable changes of cleaved caspase3/caspase3 were observed in cells with enhanced PTTG3P expression. However, over-expression of PTTG3P partially restored 5-fluorouracil-induced cleavage of caspase3 in HepG2 cells (Fig. 6d and e). Finally, we assessed the levels of several EMT-related regulators by qRT-PCR and western blot analysis. Inhibition of PTTG3P elevated the mRNA and protein levels of epithelial marker E-cadherin, but reduced the expression of mesenchymal markers Snail and Slug (Fig. 6f, g and h). Conversely, significant increases in the levels of Snail and Slug and decrease of E-cadherin were shown in cells over-expressing PTTG3P (Fig. 6f, g and h). Surprisingly, the mRNA and protein levels of mesenchymal marker N-cadherin did not display any measurable changes in cells with either stably elevated or suppressed PTTG3P expression (Fig. 6f, g and h).

Accumulating evidence supports a crucial role of PI3K/AKT signaling on regulating cell cycle progression, cell apoptosis and metastasis [19, 20]. Phosphorylated levels of PI3K and AKT were increased in cells over-expressing PTTG3P and decreased in cells with down-regulated PTTG3P compared with control cells (Fig. 6i and j).

### LncRNA PTTG3P up-regulates its parental gene PTTG1 in HCC

**Fig. 7:**
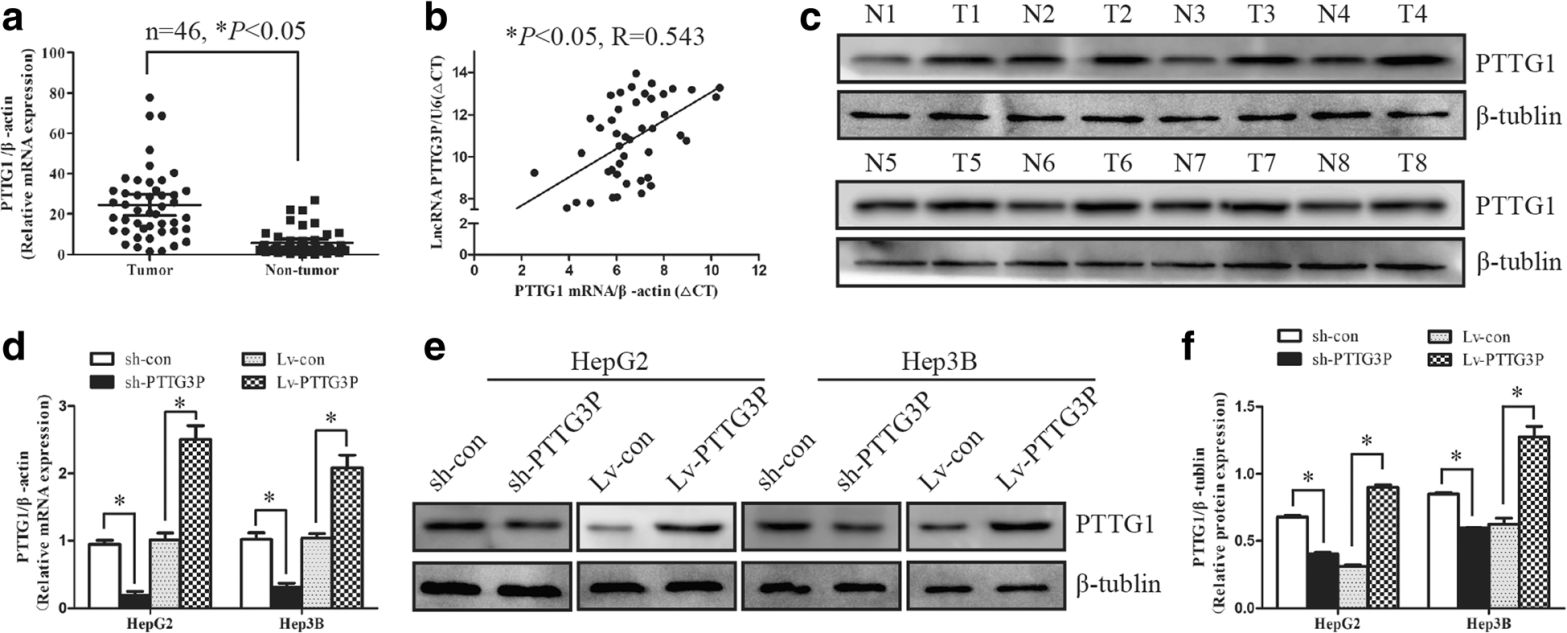
LncRNA PTTG3P up-regulates PTTG1 expression in HCC. (**a**) The level of PTTG1 was detected in 46 HCC tumor tissues and paired non-tumor tissues by qRT-PCR. β-actin was used as a housekeeping gene. (**b**) The correlation between lncRNA PTTG3P and PTTG1 mRNA in 46 HCC tissues. The ΔCt values were subjected to spearman correlation analysis. (**c**) The protein level of PTTG1 was assessed in 8 HCC tissues and paired non-tumor tissues by western blot. β-tublin was used as a loading control. T, tumor tissues; N, non-tumor tissues. (**d**-**f**) Overexpression of PTTG3P induced the expression of PTTG1 mRNA (**d**) and protein (**e**, **f**) in HepG2 and Hep3B cells. Knockdown of PTTG3P blocked PTTG1 expression (**d**-**f**). β-tublin was used as a loading control. The experiments were performed in triplicate, *P < 0.05

Previous studies have demonstrated that pseudogene can regulate parental gene expression by various mechanisms, such as microRNA sponging, chromatin remodeling [21]. Given the fact that lncRNA PTTG3P is a pseudogene-expressed non-coding RNA with high homology of its parental gene PTTG1 (Additional file 6: Figure S3), we suggested that PTTG3P could modulate the expression of PTTG1. To test our hypothesis, the levels of PTTG1 were detected in 46 pairs of HCC/non-tumor tissues (cohort 1) by qRT-PCR and western blot. Higher levels of PTTG1 were observed in HCC tumor tissues than non-tumor tissues (Fig. 7a and c). Moreover, the level of PTTG3P was positively correlated with PTTG1 mRNA in 46 HCC tumor tissues (R = 0.543, *P* < 0.05, Fig. 7b). We further clarified the regulatory relationship between PTTG3P and PTTG1 in HCC cells. Overexpression of PTTG3P markedly induced PTTG1 expression whereas knockdown of PTTG3P significantly blocked the expression of PTTG1, suggesting that PTTG3P could up-regulate PTTG1 expression (Fig. 7d, e and f).

## Discussion

PTTG3P, also known as PTTG3 or rcPTTG1, was firstly identified by Leilei Chen and his colleagues in 2000 [22]. They reported that PTTG3P is located at chromosome 8q13.1 and show high homology to hPTTG gene through Southern blot analysis, Northern blot analysis, sequencing and restriction map analysis of the genomic clones. At that time, they regarded PTTG3P as a protein coding gene. However, to date, PTTG3P has been confirmed to be a known processed pseudogene rather than a protein coding gene by automated computational analysis using gene prediction method (http://www.pseudogene.org). Pseudogenes are structurally similar to genes that encode functional proteins, but contain “defects” that, in most cases, render them unable to encode fully functional proteins [21]. Recently, the attribution of function to specific pseudogenes has raised them to the status of a new class of regulatory lncRNAs involved in both physiological and pathological processes [21]. For instance, lncRNA PTENP1, a processed pseudogene of the tumor suppressor PTEN, has been demonstrated to increase PTEN abundance and then be actively involved in cancer pathogenesis [23]. Apart from PTENP1, examples include FTH1 pseudogenes [24], PDIA3P [25] and BRAFP1 [26]. However, few studies have investigated the role and mechanism of PTTG3P in HCC progression.

In this study, we demonstrated that PTTG3P is frequently up-regulated in HCC tissues relative to corresponding adjacent non-tumor tissues from 2 cohorts by qRT-PCR and ISH assays. Clinical data revealed that high levels of PTTG3P significantly correlate with the invasive and aggressive characteristics of HCC (positively correlated with tumor size and TNM stage) as well as poor survival in patients with HCC. These results implicated that over-expression of PTTG3P may be a common feature in HCC and might serve as a valuable prognostic biomarker for HCC. Consistent with our findings, a microarray analysis carried out by Jennen, D. G. et al. [27] revealed that compared with control cells, higher levels of PTTG3P is observed in HepG2 cells exposed to aflatoxin B1 for 48 h. Aflatoxin B1 exposure is a well-known risk factor for HCC [28, 29]. Moreover, higher level of PTTG3P has been reported to be correlated with shorter disease-free survival and overall survival in patients with gastric cancer [30]. Thus, the meaning of PTTG3P in HCC pathophysiology is strongly suggested.

To further study the biological function of PTTG3P in HCC, we firstly performed gain-of-function and loss-of-function experiments in HepG2 and Hep3B cells. Our results showed that stably decreased expression of PTTG3P converts HepG2 and Hep3B cells into less aggressive cells, with higher capability of cell apoptosis and lower capability of cell-cycle G1/S transition, cell growth in vitro and tumorigenicity in vivo, cell migration and invasion in vitro as wells as metastasis in vivo. In contrast, elevated PTTG3P expression has opposite effects. Thus, these data indicated that PTTG3P functions as an oncogene in HCC. Similar to our results, a genome-scale RNAi profiling of cell division in human tissue culture cells showed that siRNA specially targeting PTTG3P results in G0/G1 arrest [31]. Moreover, Kho, P. S et al. [32] reported that 5-fluorouracil results in decreased level of PTTG3P, partly in support to our finding that over-expression of PTTG3P partially restores 5-fluorouracil-induced cell apoptosis. Consistently, PTTG3P has been demonstrated to promote gastric tumor cell proliferation and invasion [30].

The biological roles of PTTG3P found in this study provide a mechanistic basis in HCC. It has been well-defined that unscheduled proliferation is often induced by cell cycle defects as well as dysregulation of cyclins and cyclin-dependent kinases (CDKs) complexes [33]. Here, we showed that the molecular mechanisms of PTTG3P on promoting cell proliferation might be the acceleration of G1/S transition, elevated expression of C-myc, CyclinD1and p-Rb under enhanced expression of PTTG3P. CyclinD1, a key G1 phase-associated cell cycle regulator, is required for hyperphosphorylation of Rb and dissociation of transcription factor E2F from Rb, leading to the transcription of genes implicated in S phase progression [34]. Moreover, a bulk of findings reported that C-myc over-expression stimulates cell cycle progression by targeting multiple genes related to cell cycle control including Cdc25A, CyclinE1 as well as Cyclin D1 [35]. However, whether PTTG3P up-regulates Cyclin D1 via activation of C-myc need further investigation. Intriguingly, CDK4 and CDK6 are not involved in cell proliferation induced by PTTG3P. In addition to enhanced proliferation, resistance to apoptosis is also a hallmark of cancer cells [18]. In current study, PTTG3P down-regulation activated caspase3 whereas PTTG3P over-expression partially abrogated 5-fluorouracil-induced activation of caspase3. Although PTTG3P has been linked to regulation of metastasis, the molecular mechanisms remain poorly elucidated. EMT, which is characterized as a down-regulation of epithelial markers, particularly E-cadherin, and an up-regulation of mesenchymal markers, particularly N-cadherin, snail and slug, is a crucial step for cancer invasion and metastasis in various cancer cells [36, 37]. Here, more specifically, our data revealed that depletion of PTTG3P inactivates EMT processes by up-regulation of E-cadherin and down-regulation of snail and slug. However, N-cadherin is not implicated in EMT induced by PTTG3P.

PI3K/AKT pathway, activated in 40–50% of HCCs, plays a crucial role in the cell growth and metabolism ultimately influencing the invasion, metastasis and aggressiveness of cancer cells [20, 38]. Once activated, PI3K/AKT signal could increase the levels of C-myc and CyclinD1, thereby promoting G1/S transition and cell proliferation [39]. Moreover, previous study has revealed that inhibition of PI3K/AKT induces apoptotic and autophagic cell death [40, 41]. Additionally, accumulating evidence supports a vital role of PI3K/AKT pathway on EMT and metastasis by down-regulating E-cadherin as well as up-regulating Snail and Slug [40].Currently, studies have identified several lncRNAs to function through PI3K/AKT signaling [41]. Here, we showed that PI3K/AKT pathway was activated since p-PI3K and p-AKT were up-regulated by PTTG3P. Therefore, the up-regulation of C-myc, CyclinD1, Snail and Slug as well as down-regulation of E-cadherin by PTTG3P probably results from enhanced PI3K/AKT pathway activity. This thus explains the induction of cell growth and metastasis by PTTG3P in HCC.

Accumulating evidence suggest that pseudogenes play critical roles in various diseases through regulating parental gene expression [42]. Examples include PTENP1 and its parental gene PTEN, OCT4-pg4 and its parental gene OCT4 [42].Since PTTG3P is a known processed pseudogene, we found PTTG1 to be a parental gene of PTTG3P. PTTG1 was reported to be up-regulated in various cancers [43]. Overexpression of PTTG1 can promote cell proliferation, inhibit cell apoptosis and induce EMT [44, 45]. Moreover, PTTG1 has been demonstrated to activate PI3K/AKT signaling [46]. Thus, we hypothesized that PTTG3P could activate PI3K/AKT signaling and promote HCC progression by regulating PTTG1. To test our hypothesis, the regulatory relationship between PTTG3P and PTTG1 in both tumor tissues and HCC cells was further clarified. We revealed that PTTG1 was frequently up-regulated and positively correlated with PTTG3P in 46 HCC tumor tissues. Overexpression of PTTG3P resulted in up-regulation of PTTG1 whereas knockdown of PTTG1 inhibited PTTG1 expression. Our results suggest that PTTG1 could be induced by PTTG3P. Consistently, the microarray data in Oncomine database (http://www.oncomine.org) support a positive correlation between PTTG3P and PTTG1 in ovarian serous adenocarcinoma tissues, breast carcinoma tissues and rectal adenocarcinoma tissues. However, the study carried out by Weiwei Weng et al. [30] found PTTG3P expression is independent of PTTG1 in both gastric cancer tissues and cells. It seems that the relationship between PTTG3P and PTTG1 varies in different cancers. Nevertheless, a limitation of this current study is that we did not demonstrate a direct molecular function of PTTG3P in up-regulating PTTG1, and further investigation is needed.

## Conclusions

**Fig. 8:**
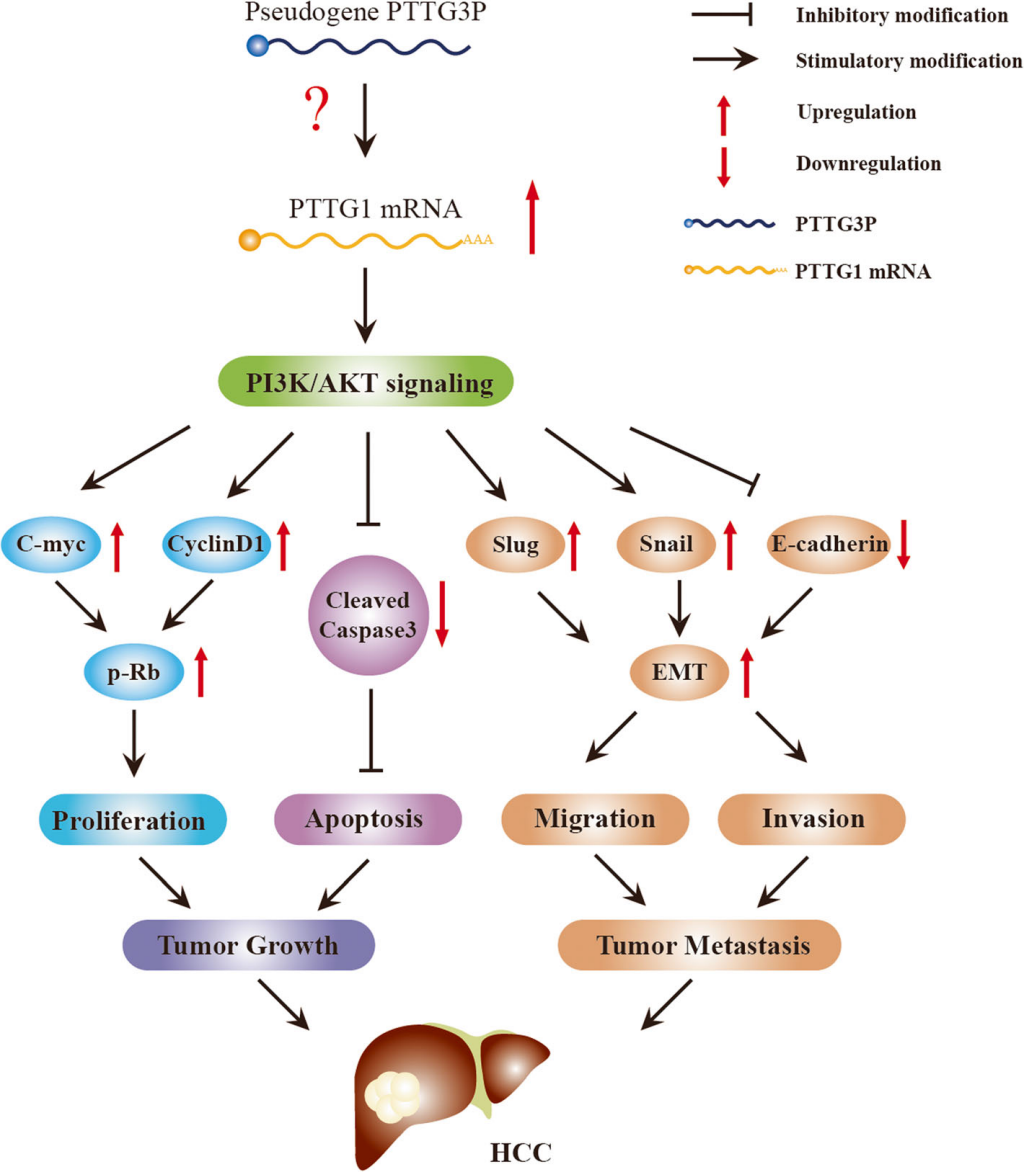
A schematic model of lncRNA PTTG3P functions during tumor growth and metastasis cascade in HCC. LncRNA PTTG3P up-regulates PTTG1 and then activates PI3K/AKT signaling. Activation of PI3K/AKT signaling elevates the expression of C-myc and CyclinD1, then increasing the level of phosphorylated Rb, and finally promoting cell proliferation in HCC. On the other hand, activation of PI3K/AKT inactivates caspase3 and thus inhibits cell apoptosis, thereby accelerating tumor growth. In addition, PTTG3P promotes HCC cell migration and invasion by activating PI3K/AKT signaling, up-regulating Snail and Slug, down-regulating E-cadherin, then inducing EMT

In summary, we provide first evidence that up-regulation of PTTG3P might be a valuable prognostic marker for HCC. Altered expression of PTTG3P could be important for tumorigenesis and progression of HCC. We draw a schematic model of lncRNA PTTG3P functions during tumor growth and metastasis cascade in HCC (Fig. 8). Briefly, PTTG3P promotes cell growth and metastasis via up-regulating PTTG1 and activating PI3K/AKT signaling. This in turn influences its downstream signals including multiple cell-cycle regulators and EMT-related factors in HCC. Modulation of the tumor growth and metastasis effect through inhibiting PTTG1 up-regulation and PI3K/AKT activation mediated by PTTG3P suppression might be used as a potential target for HCC prevention and therapy.

## Additional files

Additional file 1: **Table S1.** Sequences of primers and shRNA used in this study. (DOCX 29 kb)

Additional file 2: **Table S2.** The raw data for microarry analysis in 3 HCC tumor tissues and adjacent normal tissues. (XLS 13300 kb)

Additional file 3: **Figure S1.** (a) Taking the differentially expressed mRNAs in our microarray analysis as input, the pathway analysis revealed that cell cycle was the most affected biological process. (b) The level of PTTG3P in LO2, HepG2, Huh7, MHCC-97H, SMMC-7721, QGY7701, SK-Hep1 and Hep3B cells was evaluated by qRT-PCR. Relative high levels of PTTG3P were observed in Hep3B cells while relative low levels were found in HepG2 cells. U6 was used as a housekeeping gene. (TIF 4460 kb)

Additional file 4: **Table S3.** A list of top differentially expressed lncRNAs in microarray analysis. (DOCX 41 kb)

Additional file 5: **Figure S2.** (a) H&E-stained paraffin-embedded sections obtained from xenografts established by subcutaneous transplantation with sh-con and sh-PTTG3P HepG2 cells 4 weeks after cell injection. (b) H&E-stained paraffin-embedded sections obtained from xenografts established by subcutaneous transplantation with Lv-con and Lv-PTTG3P HepG2 cells 4 weeks after cell injection. (c) Representative images of PTTG3P expression from tumor xenografts established by subcutaneous transplantation with sh-con and sh-PTTG3P HepG2 cells by ISH assays. (d) Representative images of PTTG3P expression from tumor xenografts established by subcutaneous transplantation with Lv-con and Lv-PTTG3P HepG2 cells by ISH assays. (TIF 9470 kb)

Additional file 6: **Figure S3.** (a) LncRNA PTTG3P is transcribed from human chromosome 8q13.1 while the PTTG1 gene is located at chromosome 5q33.3. (b)The sequence of PTTG1 mRNA is 95% homologous identity to that of lncRNA PTTG3P in human by nucleotide BLAST. (c)The base sequence of lncRNA PTTG3P is compared to that of PTTG1 mRNA. PTTG3P shares great similarity to PTTG1 mRNA. The mismatched members of the base pair are shown in red. (JPG 3020 kb)

## Abbreviations

5-FU: 5-fluorouracil
EMT: Epithelial–mesenchymal transition
FACS: Fluorescence-activated cell sorting
H&E: Hematoxylin and Eeosin
HCC: Hepatocellular carcinoma
ISH: In situ hybridization
lncRNA: long non-coding RNA
PI3K: Phosphatidylinositol 3-kinase
PTTG1: Pituitary tumor-transforming 1
PTTG3P: Pituitary tumor-transforming 3, pseudogene
qRT-PCR: quantitative real-time polymerase chain reaction
