# Supplementary material for: The long non-coding RNA PTTG3P promotes cell growth and metastasis via up-regulating PTTG1 and activating PI3K/AKT signaling in hepatocellular carcinoma
Source: Mol Cancer. 2018 May 26;17:93. doi: 10.1186/s12943-018-0841-x (PMC5970477; doi:10.1186/s12943-018-0841-x)
Supplement: Supplementary file 1 — Table S1. Sequences of primers and shRNA used in this study. (DOCX 29 kb) [file 12943_2018_841_MOESM1_ESM.docx]

**Table S1.** Sequences of primers and shRNA used in this study.

| qRT-PCR primers | | Sequences |
| --- | --- | --- |
| lncRNA PTTG3P | sense | GGGGTCTGGACCTTCAATCAA |
|  | antisense | GCTTTAGGTAAGGATGTGGGA |
| C-myc | sense | GTCAAGAGGCGAACACACAAC |
|  | antisense | TTGGACGGACAGGATGTATGC |
| CyclinD1 | sense | GCTGCGAAGTGGAAACCATC |
|  | antisense | CCTCCTTCTGCACACATTTGAA |
| CDK6 | sense | TCTTCATTCACACCGAGTAGTGC |
|  | antisense | TGAGGTTAGAGCCATCTGGAAA |
| CDK4 | sense | ATGGCTACCTCTCGATATGAGC |
|  | antisense | CATTGGGGACTCTCACACTCT |
| Snail | sense | ACCACTATGCCGCGCTCTT |
|  | antisense | GGTCGTAGGGCTGCTGGAA |
| Slug | sense | CGAACTGGACACACATACAGTG |
|  | antisense | CTGAGGATCTCTGGTTGTGGT |
| E-Cadherin | sense | GTCCTGGGCAGACTGAATTT |
|  | antisense | GACCAAGAAATGGATCTGTGG |
| N-Cadherin | sense | TGGACCATCACTCGGCTTA |
|  | antisense | ACACTGGCAAACCTTCACG |
| Vimentin | sense | CGAGGAGAGCAGGATTTCTC |
|  | antisense | GGTATCAACCAGAGGGAGTGA |
| PTTG1 | sense | ACCCGTGTGGTTGCTAAGG |
|  | antisense | ACGTGGTGTTGAAACTTGAGAT |
| β-actin | sense | TGGCACCCAGCACAATGAA |
|  | antisense | CTAAGTCATAGTCCGCCTAGAAGCA |
| U6 | sense | CTCGCTTCGGCAGCACA |
|  | antisense | AACGCTTCACGAATTTGCGT |
| shRNA sequences | sense | CUUCAAUCCUCUAGGCUUCTT |
|  | antisense | GAAGCCUAGAGGAUUGAAGGG |
