# Supplementary figures and images for: The long non-coding RNA PTTG3P promotes cell growth and metastasis via up-regulating PTTG1 and activating PI3K/AKT signaling in hepatocellular carcinoma
Source: Mol Cancer. 2018 May 26;17:93. doi: 10.1186/s12943-018-0841-x (PMC5970477; doi:10.1186/s12943-018-0841-x)

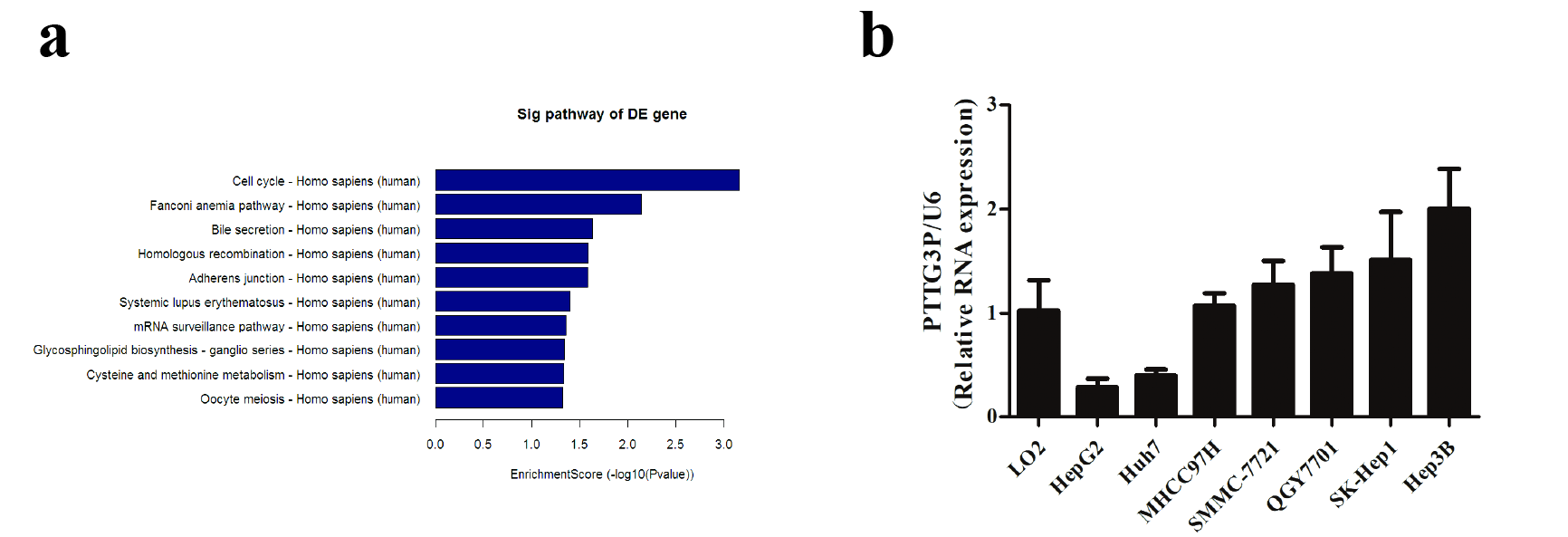

Supplement: Supplementary file 3 — Figure S1. (a) Taking the differentially expressed mRNAs in our microarray analysis as input, the pathway analysis revealed that cell cycle was the most affected biological process. (b) The level of PTTG3P in LO2, HepG2, Huh7, MHCC-97H, SMMC-7721, QGY7701, SK-Hep1 and Hep3B cells was evaluated by qRT-PCR. Relative high levels of PTTG3P were observed in Hep3B cells while relative low levels were found in HepG2 cells. U6 was used as a housekeeping gene. (TIF 4460 kb) [file 12943_2018_841_MOESM3_ESM.tif]

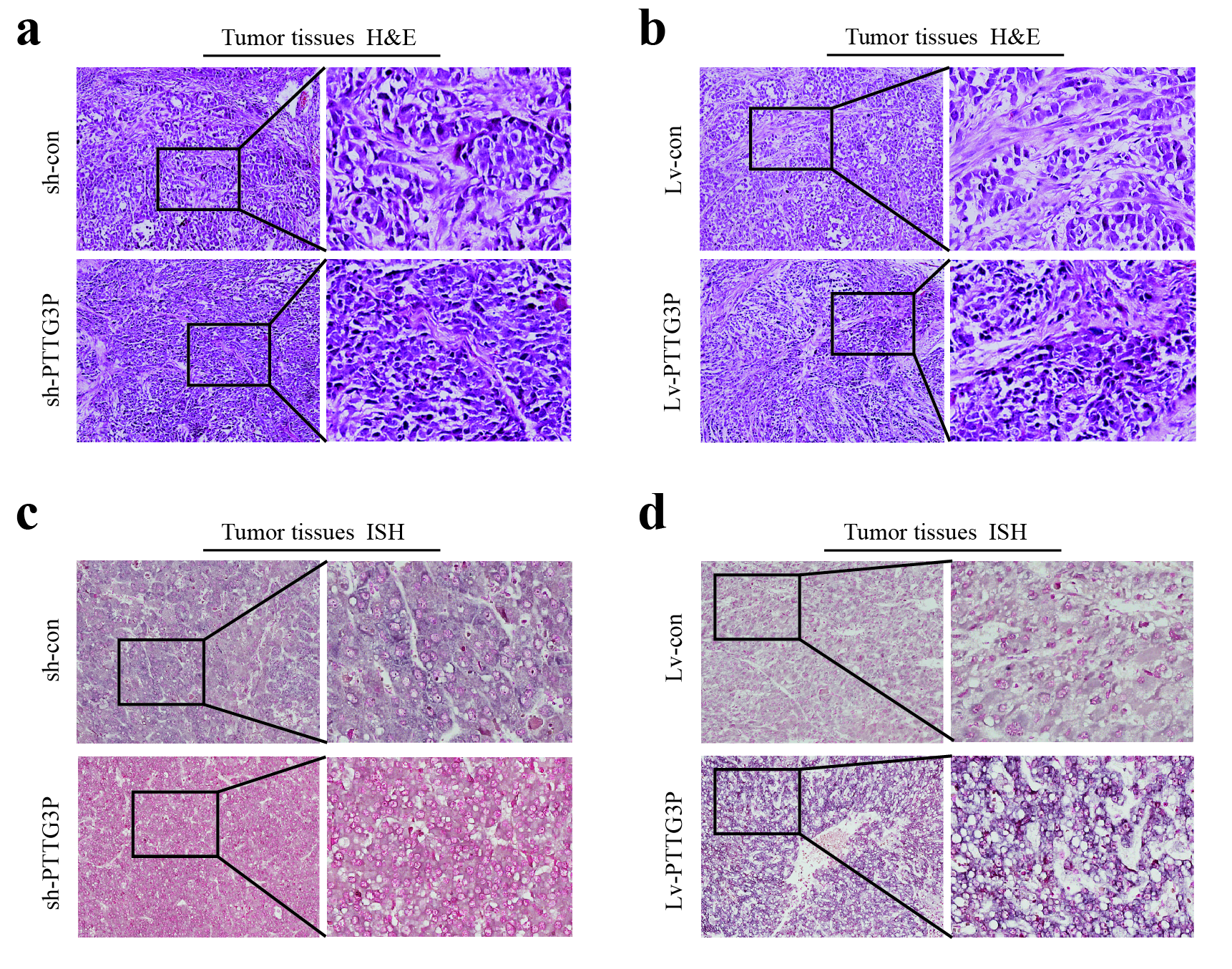

Supplement: Supplementary file 5 — Figure S2. (a) H&E-stained paraffin-embedded sections obtained from xenografts established by subcutaneous transplantation with sh-con and sh-PTTG3P HepG2 cells 4 weeks after cell injection. (b) H&E-stained paraffin-embedded sections obtained from xenografts established by subcutaneous transplantation with Lv-con and Lv-PTTG3P HepG2 cells 4 weeks after cell injection. (c) Representative images of PTTG3P expression from tumor xenografts established by subcutaneous transplantation with sh-con and sh-PTTG3P HepG2 cells by ISH assays. (d) Representative images of PTTG3P expression from tumor xenografts established by subcutaneous transplantation with Lv-con and Lv-PTTG3P HepG2 cells by ISH assays. (TIF 9470 kb) [file 12943_2018_841_MOESM5_ESM.tif]

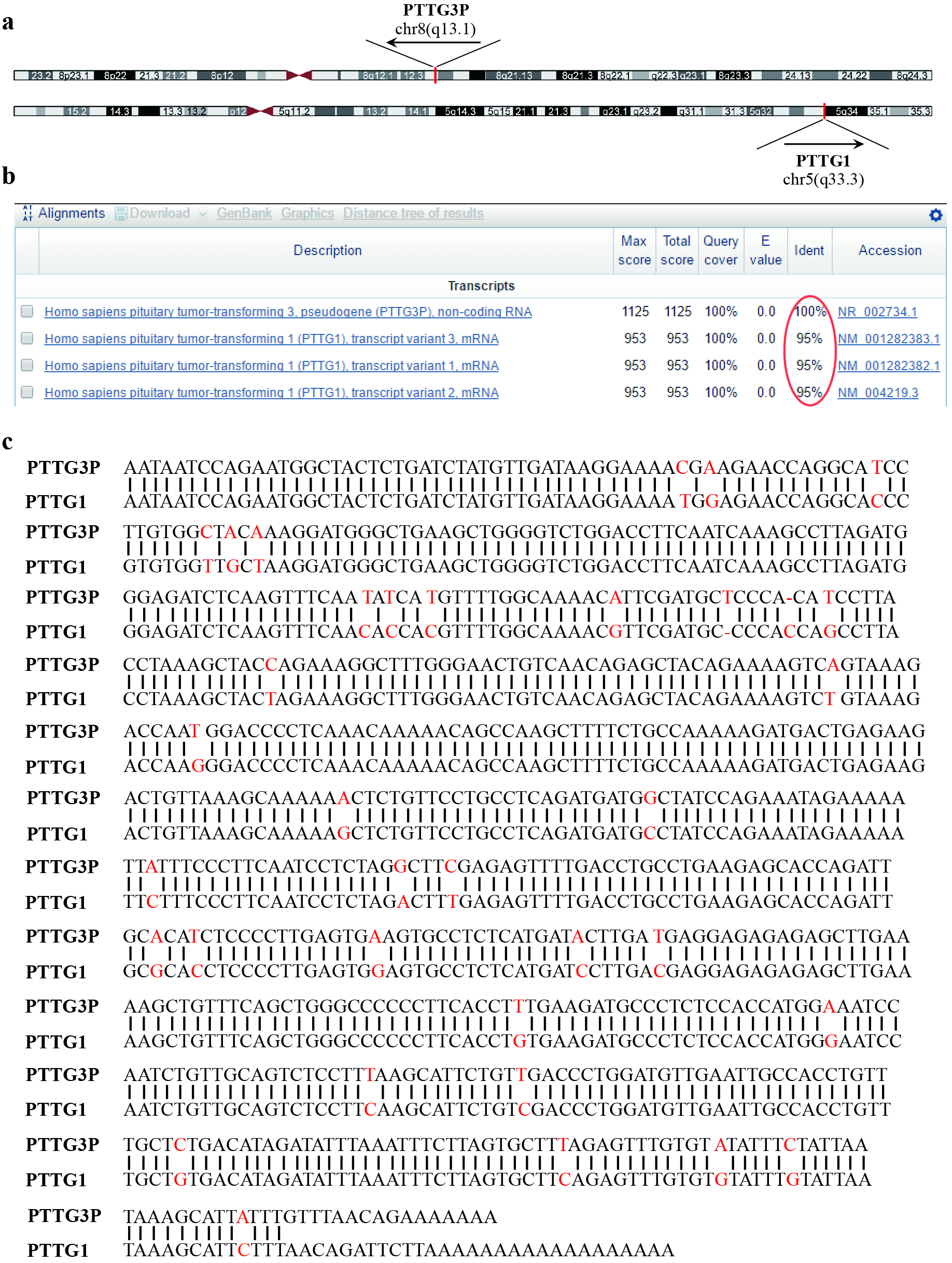

Supplement: Supplementary file 6 — Figure S3. (a) LncRNA PTTG3P is transcribed from human chromosome 8q13.1 while the PTTG1 gene is located at chromosome 5q33.3. (b)The sequence of PTTG1 mRNA is 95% homologous identity to that of lncRNA PTTG3P in human by nucleotide BLAST. (c)The base sequence of lncRNA PTTG3P is compared to that of PTTG1 mRNA. PTTG3P shares great similarity to PTTG1 mRNA. The mismatched members of the base pair are shown in red. (JPG 3020 kb) [file 12943_2018_841_MOESM6_ESM.jpg]
