# Supplementary material for: The long non-coding RNA PTTG3P promotes cell growth and metastasis via up-regulating PTTG1 and activating PI3K/AKT signaling in hepatocellular carcinoma
Source: Mol Cancer. 2018 May 26;17:93. doi: 10.1186/s12943-018-0841-x (PMC5970477; doi:10.1186/s12943-018-0841-x)
Supplement: Supplementary file 4 — Table S3. A list of top differentially expressed lncRNAs in microarray analysis. (DOCX 41 kb) [file 12943_2018_841_MOESM4_ESM.docx]

**Table S3. A list of top differentially expressed lncRNAs in microarray analysis**

| ProbeName | *P*-value | Fold change | Regulation | Gene Symbol | RNA length | chromosome | strand |
| --- | --- | --- | --- | --- | --- | --- | --- |
| ASHG19A3A037531 | 0.038 | 20.995 | up | RP11-154P18.4 | 1746 | chr9 | - |
| ASHG19A3A025588 | 0.039 | 19.038 | up | RP11-25H12.1 | 754 | chr4 | + |
| ASHG19A3A011314 | 0.001 | 16.381 | up | AURKAPS1 | 1537 | chr1 | - |
| ASHG19A3A019407 | 0.019 | 16.022 | up | AP000525.8 | 4136 | chr22 | - |
| CUST_17_PI426075208 | 0.024 | 15.841 | up | nc-HOXA13-99 | 148 | chr7 | + |
| ASHG19A3A034215 | 0.000 | 15.803 | up | AC018634.9 | 401 | chr7 | + |
| ASHG19A3A052720 | 0.022 | 13.216 | up | AC104759.2 | 531 | chr15 | + |
| ASHG19A3A034094 | 0.033 | 12.390 | up | AK093987 | 2285 | chr7 | + |
| ASHG19A3A035831 | 0.020 | 11.868 | up | PTTG3P | 609 | chr8 | - |
| ASHG19A3A025558 | 0.009 | 11.583 | up | RP11-738E22.1 | 450 | chr4 | + |
| ASHG19A3A025075 | 0.008 | 60.567 | down | AK096134 | 3158 | chr4 | - |
| CUST_177_PI426075208 | 0.034 | 51.789 | down | nc-HOXD1-48 | 138 | chr2 | - |
| ASHG19A3A045962 | 0.042 | 45.444 | down | FAM99A | 1430 | chr11 | + |
| ASHG19A3A032573 | 0.012 | 37.177 | down | AC004540.4 | 508 | chr7 | - |
| ASHG19A3A052391 | 0.038 | 28.205 | down | CTA-215D11.3 | 410 | chr1 | - |
| ASHG19A3A034624 | 0.002 | 24.454 | down | AK311103 | 3978 | chr7 | + |
| ASHG19A3A031614 | 0.042 | 14.802 | down | RP11-397G17.1 | 692 | chr6 | + |
| ASHG19A3A036203 | 0.010 | 14.532 | down | LOC100133669 | 709 | chr8 | - |
| ASHG19A3A030778 | 0.015 | 11.517 | down | LPAL2 | 2041 | chr6 | - |
| ASHG19A3A038229 | 0.002 | 11.135 | down | AF161442 | 556 | chr9 | - |
